# Hemodynamic Effects of Protamine Infusion in Dogs with Myxomatous Mitral Valve Disease Undergoing Mitral Valvuloplasty

**DOI:** 10.3390/vetsci9040178

**Published:** 2022-04-08

**Authors:** Tomohiko Yoshida, Katsuhiro Matsuura, Ahmed S. Mandour, Yuki Aboshi, Shusaku Yamada, Hideki Yotsuida, Mizuki Hasegawa, Chieh-Jen Cheng, Youta Yaginuma, Momoko Watanabe, Shou Fukuzumi

**Affiliations:** 1 VCA Japan Shiraishi Animal Hospital, 4-33-2 Sayamadai, Sayama, Saitama 350-1304, Japan; tomohiko7731-yoshida@yahoo.co.jp (T.Y.); over_the_top_1987@yahoo.co.jp (Y.A.); shusaku_yamada@hotmail.com (S.Y.); m.hasegawa1295@gmail.com (M.H.); john_199328@yahoo.com.tw (C.-J.C.); yyp.gyym.myy5@gmail.com (Y.Y.); shou.fukuzumi@gmail.com (S.F.); 2 Department of Veterinary Surgery, Tokyo University of Agriculture and Technology, Fuchu 183-0054, Tokyo, Japan; momokowatanabe526@gmail.com; 3 Department of Animal Medicine, Faculty of Veterinary Medicine, Suez Canal University, Ismailia 41522, Egypt; 4 Department of Clinical Engineering, National Cerebral and Cardiovascular Center, Osaka 564-8565, Japan; yotsuida@gmail.com

**Keywords:** protamine, hypotension, norepinephrine, cardiac surgery, cardiopulmonary bypass

## Abstract

Protamine, an antagonizing agent to heparin, is indispensable for dogs undergoing cardiopulmonary bypass. Protamine-induced hypotension (PIH) during cardiac anesthesia has been reported in humans. The purpose of this study was to describe the hemodynamic effect of protamine administration in dogs during cardiac surgery in clinical cases. Study design: Retrospective, clinical, cohort study. A total of 14 client-owned dogs who suffered heart failure due to medically uncontrolled myxomatous mitral valve disease (MMVD) were included in this study. The severity of MMVD was classified according to American College of Veterinary Internal Medicine staging (ACVIM: stage B2, C, D) and dogs undergoing mitral valve surgery. Records with clinical data for dogs treated between July 2019 to August 2020 were examined for age, sex, breed, body weight, concurrent diseases, hospitalization, anesthetic record, and mortality within 3 months after the operation. PIH was defined as mean arterial pressure (MAP) lowered by 20% of that before protamine infusion. To evaluate the effect of protamine on hemodynamic variables, each of the other values was compared with values at the beginning of protamine infusion. MAP decreased by 41.0 and 45.7% in two dogs (14.3%) compared with pressure before protamine infusion. Others did not show obvious alteration in hemodynamic variables. Epinephrine treatment alleviated hypotension in one dog. Another dog with systemic hypotension concomitant with elevated central venous pressure did not respond to epinephrine treatment and a reboot of extracorporeal circulation was required. Reheparinization and reinstitution of cardiopulmonary bypass successfully resuscitate the second dog. In conclusion, clinicians should alert the incidence of severe hypotension even with slow protamine infusion following canine cardiac surgery. This study also provides two effective treatments for catastrophic hypotension during protamine infusion.

## 1. Introduction

Myxomatous mitral valve disease (MMVD) resulting from degeneration of the valve leaflets and chordae tendineae is a common cardiac disorder causing congestive heart failure in dogs, especially in small breeds [1,2]. Surgical intervention for the advanced stages of canine MMVD is now available in some countries because of recent advances in surgical procedures [2,3,4,5]. In particular, mitral valvuloplasty (MVP) has been considered a treatment option for MMVD. MVP is performed under the cardiopulmonary bypass in which heparin is necessary to prevent thrombus formation.

Protamine is commonly used to reverse the anticoagulant properties of heparin after cardiac surgery. However, hemodynamic instability due to protamine is one of the critical complications in cardiac surgery since the administration of protamine is associated with systemic hypotension, which may sometimes be fatal [6,7]. Protamine-induced hypotension (PIH) occurs when heparin is antagonized after the end of cardiopulmonary bypass. Protamine causes negative inotropic and systemic vasodilator effects in humans and experimental dogs [8,9]. According to reports on experimental dogs, 65% experienced systemic hypotension [10]. In another study, hypotension is less likely to occur when injected slowly than with rapid administration in dogs [11].

Since PIH has been reported in experimental dogs, hypotension may occur when used in clinical cases. However clinical manifestation of protamine reaction is not demonstrated in veterinary medicine. The present study aimed to describe the hemodynamic effect of protamine injection following mitral valve surgery in dogs with spontaneous degenerative mitral valve disease.

## 2. Materials and Methods

### 2.1. Animals

Fourteen dogs that suffered from MMVD and underwent mitral valvuloplasty were retrospectively evaluated in this study. Data were collected from the medical and anesthetic records of a private clinic specialized in veterinary cardiology (SHIRAISHI Animal Hospital, Saitama, Japan) between July 2019 and August 2020. All dogs were monitored for 3 months after the repair of spontaneous mitral valve regurgitation. The severity of mitral valve disease was staged according to the American College of Veterinary Internal Medicine (ACVIM) consensus statement guidelines by Keene et al. [1]. Inclusion criteria for surgery included a diagnosed Myxomatous mitral valve disease (MMVD), an ACVIM stage of at least B2. Two had been prescribed an angiotensin-converting enzyme inhibitor (ACEI), fourteen had been prescribed pimobendan, ten dogs had been prescribed furosemide, five had been prescribed torsemide, and five had been prescribed spironolactone. The clinical diagnosis, staging, and decision for the surgical indication of MMVD were determined through comprehensive evaluation including medical history recoding, physical examination, hematology, biochemistry, diagnostic electrocardiography, radiography, and echocardiography.

### 2.2. Echocardiography

To evaluate the severity of MMVD, conventional echocardiography protocol (two-dimensional, M-mode, Doppler blood flow, and Tissue Doppler imaging techniques) was carried out [12,13]. Dogs were positioned in right- and left-lateral recumbency without anesthesia. The echocardiographic assessment was performed using Aplio 300 (Canon medical system, Tokyo, Japan), with a 5-MHz sector probe at a sweep speed of 200–300 mm/s and a sample gate of 1.5 mm. The following echocardiographic variables associated with the ACVIM stage were measured: from the right parasternal short-axis view at the mid-papillary muscle level, left ventricular internal diameter in diastole (LVIDd) and fractional shortening (FS) were measured. In the short-axis view at the aorta level, the ratio between the left atrium and the aortic diameter (Ao), was measured (LA/Ao). The left apical four-chamber view was optimized for the evaluation of the transmitral flow and tissue Doppler imaging (TDI). The early diastolic mitral inflow (E velocity), late diastolic mitral inflow (A velocity), and the ratio between early and late velocities (E/A) were assessed using Pulsed-wave Doppler echocardiography. In addition, systolic (S’), early diastolic (E’), and late diastolic (A’) annular tissue velocity waves were measured by focusing the sample volume of the TDI at the septal (sep) and the left ventricular lateral (lat) wall, respectively. LVIDDN was calculated using an established allometric formula [12].

### 2.3. Surgical Procedure

Fourteen dogs were premedicated with atropine sulfate 0.05 mg kg^–1^ SC (Atropine Injection; Tanabe Seiyaku Co., Ltd., Osaka, Japan), fentanyl 5 μg kg^–1^ IV (Fentanyl Injection; Daiichi-Sankyo, Tokyo, Japan) and midazolam 0.2 mg kg^–1^ IV (Midazolam Injection; Takeda Pharmaceutical Co., Ltd., Osaka, Japan), followed by induction with propofol 6 mg kg^–1^ IV (Propofol 1% Injection; Pfizer Japan Inc., Osaka, Japan), and maintained with 1–2 vol% of isoflurane (Isoflurane Inhalation Solution; Pfizer Japan Inc., Osaka, Japan) and propofol. Methylprednisolone (Methylprednisolone Sodium Succinate, Pfizer Japan Inc., Japan) and cefazolin (Cefazolin Sodium Injection, LTL Pharma Co., Ltd., Tokyo, Japan) were injected as anti-inflammatory and antibiotics, respectively. Fentanyl (10 μg^–1^ kg^–1^ h) was used as intra operative analgesia. A 4-Fr catheter (Atom nutrition catheter, Atom Medical, Tokyo, Japan) was inserted into the right femoral artery and vein to monitor systolic (SAP), mean (MAP), diastolic (DAP) arterial pressures, and central venous pressure (CVP). Then, these catheters were connected to pressure transducers (Life kit DX-360, Nihon Kohden, Tokyo, Japan) and pressure information was imaged by a multi-channel monitor (Life Scope BSM-5192; Nihon Kohden, Tokyo, Japan). Heart rate was measured by electrocardiogram. Heparin (Heparin Sodium Injection; Nipro, Tokyo, Japan) 400 U kg^–1^ was administered. To perform cardiopulmonary bypass (CPB), the left carotid artery and jugular vein were cut down with a No. 11 scalpel and an 8–14 Fr catheter (Bio-Medicus NextGen, Medtronic Japan Co., Ltd., Tokyo, Japan) was inserted according to the dog’s size. CPB (D100 KIDS artificial lung, LivaNova Japan, Tokyo, Japan) with a flow of rate 100 mL^−1^ kg^–1^ min was used. Activated clotting time (ACT+; Accriva Diagnostics, San Diego, CA, USA) was used for determining the anticoagulant effect of heparin. Aortic root cannula was performed through a purse-string suture from the left lateral side of the ascending aorta. Aortic clamping followed by the administration of cardioplegia (Miotecter; Fuso Pharmaceutical Industries, Ltd., Osaka, Japan) achieved cardiac arrest. The mitral valve was repaired by chordal reconstruction and suture annuloplasty. After the closure of the left atrium, the aorta was unclamped and sinus rhythm was restored spontaneously. Dog weaned from CPB after conducting modified ultrafiltration and confirming the hemodynamic system was stable. Ultrafiltration was performed by previously reported methods [14]. After weaning from CPB, a constant rate infusion via a cephalic vein catheter of protamine (Protamine Sulfate Injection; Mochida Pharmaceutical Co., Ltd., Tokyo, Japan) 4 mg kg^–1^ was administered over 30 min using a syringe pump. Norepinephrine and/or dobutamine were administered as needed. Activated clotting time (ACT) was measured after the end of protamine infusion. The surgical procedure is summarized in Supplementary Figure S1.

In the immediate recovery period, IV fluid therapy including fentanyl was administered and monitoring was performed using an electrocardiogram (Life Scope BSM-297 5192; Nihon Kohden, Tokyo, Japan), pulse oximeter (Life Scope BSM-297 5192; Nihon Kohden, Tokyo, Japan), and a pressure transducer (Life kit DX-360, 295 Nihon Kohden, Tokyo, Japan). Invasive blood pressure was measured from dorsalis pedis artery.

### 2.4. Definition of Hypotension after Protamine Infusion

PIH was defined as mean arterial blood pressure (MAP) lowered by 20% of that before protamine infusion for five min [15]. Definition of hypotension: 

MAP nadir during protamine infusion < MAP before protamine infusion × 0.8.

### 2.5. Protocol of Protamine and Catecholamine Administration

Protamine 4 mg kg^–1^ was administered over 30 min. If significant hypotension occurred during protamine administration, protamine administration was discontinued and doses of norepinephrine and dobutamine were started to deal with hypotension. Norepinephrine and dobutamine were started from 0.1 μg^–1^ kg^–1^ min and 3.0 μg^–1^ kg^–1^ min, respectively. When MAP recovered to the value before the administration of protamine, protamine administration was resumed. CPB was started when the administration of protamine induced severe hypotension which could not be dealt with the catecholamine.

Generally, if hypotension was not improved, the dose of dobutamine and norepinephrine was gradually increased every 5 min. Dosing of norepinephrine (first dose 0.1 then 0.2 μg^–1^ kg^–1^ min) and dobutamine (first dose 3.0 then 5.0 then 7.0 then 10 μg^–1^ kg^–1^ min) were followed.

### 2.6. Animal Classification

According to the effect of protamine on blood pressure, dogs were classified into two groups as follows; a non-hypotensive group (blood pressure remain not significantly reduced (*n* = 12) and a hypotensive group where the blood pressure was reduced by 20% after protamine administration (*n* = 2).

### 2.7. Statistical Analysis

The significance level was set at *p* < 0.05. One-way analysis of variance for repeated measures followed by a Dunnett’s test, comparing each of the other values to values at the beginning of protamine infusion, was used to evaluate the effect of protamine on hemodynamic variables. Statistical analyses were performed using R software (Version 3.3.2).

## 3. Results

### 3.1. Characteristics of Dogs and Preoperative Echocardiography Examination

Table 1 shows the clinical characteristics, ACVIM stage of heart failure, concurrent disease, and perioperative information of the investigated dogs. Nine dogs were male and five were female. In the non-hypotensive group, Chihuahua and Mongrel represent 41.7% (5/12) and 16.7 (2/12), respectively; meanwhile, other dog breeds represented only one dog. In the non-hypotensive group, based on ACVIM classifications, 41.7% (5/12) of dogs were at ACVIM stage B2, 33.3% (4/12) of dogs at stage C, and 25% (3/12) were at stage D. The two dogs in the hypotensive group were at ACVIM stage C. Renal failure, epilepsy, hydrocephalus, hypothyroidism, and idiopathic thrombocytopenia were reported as concurrent diseases in the non-hypotensive group (one dog for each disease). Meanwhile, no concurrent disease was reported in the hypotensive group.

**Figure 1:**
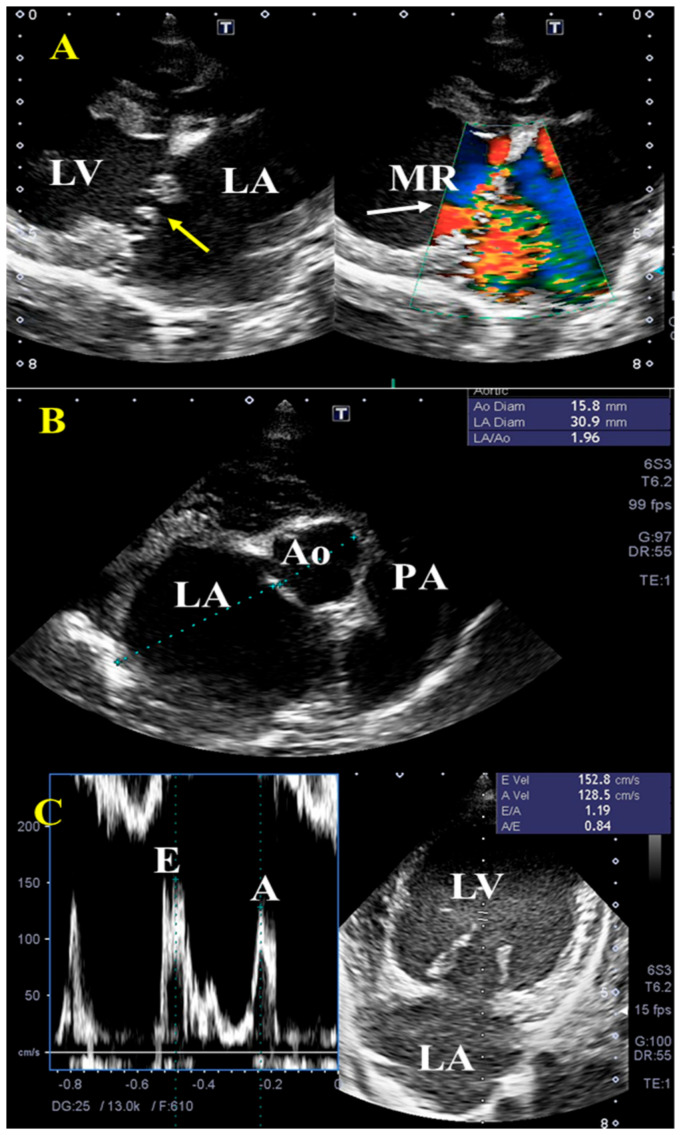
Representative transthoracic echocardiographic findings in dogs with myxomatous mitral valve disease (MMVD). Right parasternal long-axis view (**A**) shows thickening of the mitral leaflets (yellow arrow), enlarged left ventricle (LV), and left atrium (LA), with concurrent mitral regurgitation (MR, white arrow, grade C to D according to ACVIM). The increased left atrium diameter to aortic diameter ratio (>1.5) (**B**) as well as early velocity (E) of the mitral inflow (**C**) were observed. PA, pulmonary artery.

The result of preoperative echocardiographic variables was shown in Table 2. Figure 1 is representative echocardiogram images of MMVD dogs in this study. In all dogs, left atrium enlargement, mitral regurgitation, and an increase in E wave peak velocity were found on standard echocardiographic views (Figure 1A–C).

### 3.2. Dose of Catecholamines during Protamine Infusion

Table 3 illustrates the dose of catecholamines and dobutamine during operation. Constant rate infusion of norepinephrine at a mean dosage of 0.05 μg^–1^ kg^–1^ min was given throughout protamine infusion (0.0–0.1 μg^–1^ kg^–1^ min) to the non-hypotensive dogs. The dose of norepinephrine was a two-fold and four-fold increase in the two hypotensive dogs, respectively, compared with the mean dose in the non-hypotensive dogs. Simultaneously, dobutamine was used at a mean dosage of 4 μg^−1^ kg^−1^ min in the non-hypotensive group which was increased to 5 μg^–1^ kg^–1^ min in the two hypotensive dogs.

### 3.3. Arterial and Central Venous Pressure after Protamine Infusion

**Figure 2:**
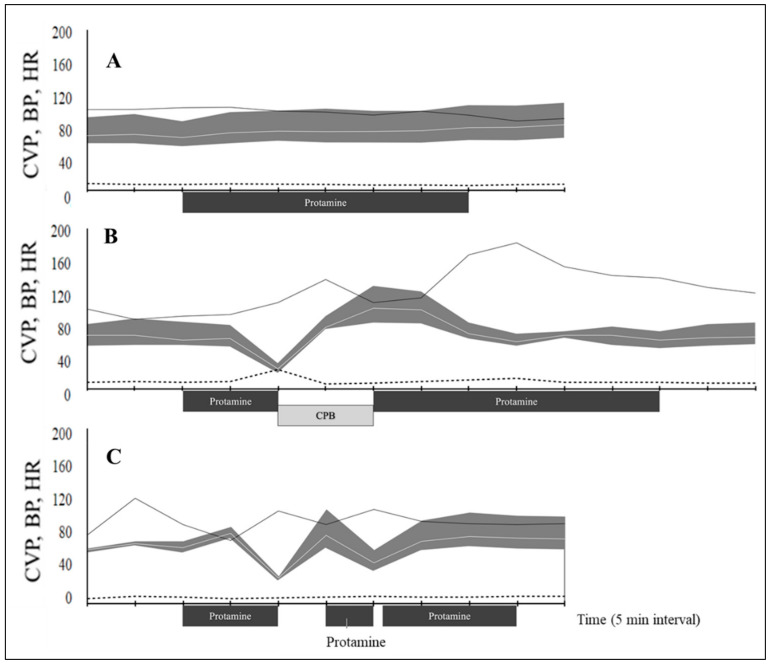
Hemodynamic variables during protamine infusion in dogs undergoing cardiopulmonary bypass for mitral valve repair. (**A**) Mean values for 12 dogs without hypotension. Dog number 1 (**B**) and dog number 2 (**C**) show protamine shock. The blood pressure (BP, mmHg) is presented as a gray shading curve. Mean arterial pressure (white line within grey shading); the upper and lower limits of the shading curve are systolic and diastolic pressures); heart rate (HR, bpm; solid black line); and central venous pressure (CVP, mmHg; dashed line) recorded every 5 min from 10 min before the start of protamine administration. CPB, cardiopulmonary bypass.

Dogs were classified into two groups based on their response to protamine administration. In the non-hypotension group, there was no significant change in heart rate, systolic arterial, diastolic arterial mean arterial, and central venous pressure (CVP) during protamine infusion (Table 4 and Figure 2A). The CVP was approximately the same in all dogs (8 mmHg) except in hypotensive dog number one which showed a significant increase in CVP (18 mmHg) after protamine infusion (Figure 2B). 

### 3.4. Protamine-Induced Hypotension

The adverse reaction of protamine in this study was observed as hypotension. PIH was observed in 2 out of 14 dogs (14.3%). The two dogs with PIH exhibited marked signs 10 min after the infusion. In one dog, MAP suddenly decreased from 63 to 25 mmHg for 5 min. MAP nadir was 41.0% compared with the pressure before infusion. Administration of epinephrine at a dose of 10 μg^–1^ kg repeatedly failed to restore cardiovascular collapse. Visually, the myocardium lost its dynamism and hemoglobin saturation. We decided the reboot extracorporeal circulation to avoid life-threatening damage to systemic organs by prolonged hypotensive state. Heparin 400 U^–1^ kg was added to the extracorporeal circuit before the second cardiopulmonary bypass. After hemodynamic support by an artificial heart-lung system, the heart regained cardiac output, and the blood pressure became stable. A total of 4 mg^–1^ kg protamine was infused after the end of the second cardiopulmonary bypass (Figure 2B). In the second dog, MAP decreased to 46% of the preinfusion value within 5 min after starting the protamine infusion. One-time IV bolus of epinephrine 10 μg^–1^ kg stabilized MAP to ≥80 mmHg within 5 min. The rest of the protamine was administered at half the initial rate once hemodynamic variables were stabilized. The second administration of protamine resulted again in decreased pressure; however, norepinephrine was effective in stabilizing the blood pressure. After blood pressure was improved, protamine was restarted (third dose). By the third dosing of protamine, administration of all the prescribed amounts was completed (Figure 2C).

### 3.5. Activated Clotting Time (ACT)

**Figure 3:**
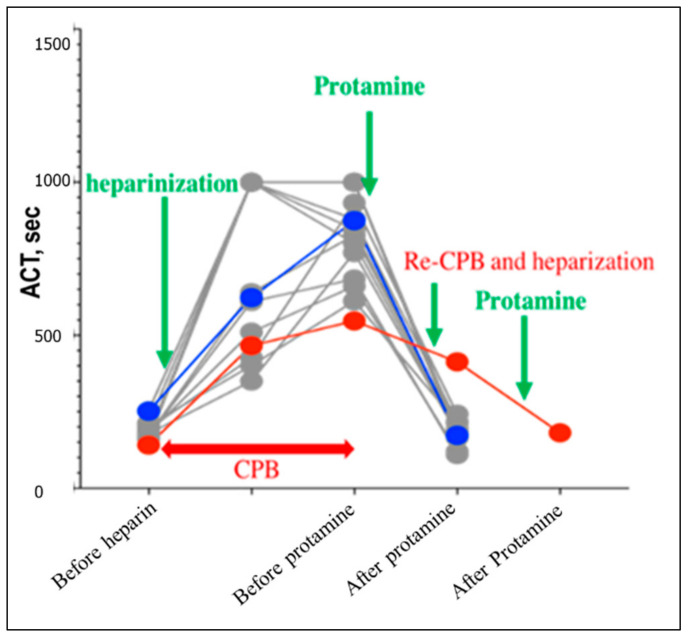
Plotting of the activated clotting time (ACT) throughout the cardiopulmonary bypass of dogs undergoing mitral valvoplasty. Gray dots resemble dogs from the non-hypotensive group. Hypotensive dog number one and dog number two were dotted red and blue, respectively. The examination time was set before heparinization, before the first protamine, and before the second protamine administration in all dogs. In hypotensive dog number two, the ACT was evaluated also after heparinization.

Data of ACT are summarized in Figure 3. The median ACT was 186.5 s (range: 141–308) in non-hypotensive dogs, and 167 s and 252 s in hypotensive dog number 1 and dog 2, respectively. By the first protamine infusion, ACT was increased by 834 s (361–1000 <), 430, and 850 s; respectively in the same order. After the second protamine, ACT values were greatly decreased in all dogs. After heparinization of dog number 2, the ACT was 413 s.

### 3.6. Outcome

One dog from the non-hypotension group died 6 days after surgery due to cerebral infarction. Two dogs with hypotension did not experience any complications. The overall mortality rate was 7.1% (1/14) at 3 months after surgery.

## 4. Discussion

MMVD is the most common cardiac disorder in dogs where surgical interference remains the last resort when medical treatment is ineffective. Despite the invasiveness and long operation time, the successful rate of mitral valvoplasty through open-heart surgery is still the best among other choices. This study demonstrated a clinical response to protamine infusion following open-heart surgery and strategies to combat PIH to enhance the recovery and the outcomes of patients. Clinical and experimental studies demonstrated that the usage of protamine as a heparin-neutralizing agent has a negative impact. This includes increased pulmonary artery pressures, decrease systemic BP, increases myocardial oxygen consumption, increase cardiac output and heart rate, and decreases systemic vascular resistance [16].

Sudden catastrophic hypotension occurred 10 min after the onset of infusion. Epinephrine bolus and reboot of extracorporeal circulation were useful in solving the hemodynamic perturbations. In the current study, two out of 14 dogs experienced a reduction in MAP of more than 20% compared to the pre-infusion period, and 12 out of 14 operated dogs showed no deterioration in the cardiovascular system and did not require additional use of vasoactive medication. In the two affected cases, the sudden cardiovascular collapse may be induced by protamine. As previously observed in other studies in humans [7,17], hypotension in the two cases was observed within 10 min from the beginning of protamine infusion. Additionally, the severity (ACVIM stage) of the case was not considered to be related to hypotension. Thus, the factor that triggers the onset of hypotension by protamine remains unknown. Mechanisms of adverse protamine reactions are the direct suppression of myocardial contractility, systemic vasodilation, and pulmonary vasoconstriction [11,15,18,19]. The remarkable elevation of central venous pressure in dog 1 (18 mmHg) can be associated with pulmonary hypertension concomitant with severe systemic hypotension, but this was not the case in dog 2 hence venous pressure elevation was not observed in the other dog (8 mmHg). This study provides some information regarding the variability of adverse protamine reactions in dogs following cardiac surgery. It is worth noting that PIH can develop at a low rate of 0.13 mg/kg/min in this study. In experimental dogs, the rapid infusion of 10 mg/kg/min greatly inhibits the circulatory system compared to a slow administration rate of 2.5 mg/kg/min [10,11]. Adverse hemodynamic reactions of protamine in humans are also less severe with a slow rate infusion for 15 min than with a 3 min method [20]. Slow rates appear to contribute to the decreased incidence; however, it remains unclear how slow the drug should be given. Furthermore, depression of the circulatory system was also observed in the second dose in hypotensive dog number 2. The second administration was a further slower rate of 1/2 than the first. Therefore, slowing the administration rate alone may be an incomplete strategy to cope with this condition.

This study presented two effective treatments for PIH. Epinephrine has been used in humans for treating protamine side effects and is suitable as a first choice [21]. The administration of epinephrine has repeatedly shown success in resolving hypotension. However, some dogs are non-responsive to epinephrine treatment which may result in death because of hypotensive crisis, as previously reported in human studies [6,7]. In hypotensive dog number 1, the hypotension did not resolve despite multiple administrations of epinephrine. Adverse events associated with hypotension observed by protamine administration are divided into some patterns. The most frequent pattern is shock due to protamine hypersensitivity [22,23]. The protein contained in protamine may cause histamine-mediated venous dilatation. Although the details are not clear, hypotensive dog number 1 may have been shocked in response to the protein contained in protamine. In this dog, the second extracorporeal hemodynamic support was useful for saving a life. Reheparinization and reinstitution of cardiopulmonary bypass can be a treatment of choice for PIH in dogs as in humans [24].

The description of PIH was discussed extensively in this study. Protamine infusion suddenly causes life-threatening hypotension without warning. Protamine infusion must be given slowly and carefully monitored until the end of the administration. Since there is no established treatment, the worst scenario could be avoided by maintaining the cannula in the dog, so that the extracorporeal circulation can be resumed at any time until the end of protamine infusion. To prevent clotting in the extracorporeal circuit, heparin should be added to the circuit and the blood is circulated by using the shunt in the circuit. In human medicine, several studies reported that the administration of methylene blue may be effective for PIH; however, the effect of this drug is still skeptical in veterinary medicine [16]. 

The small sample size is one of the major limitations for an accurate assessment of incidents, risk factors, and prognosis. Large-scale studies can help identify true prevalence and risk factors. Although re-pump can be the last of the bastion to save a life, this method has a limitation of invasiveness. According to human literature, prolonged cardiopulmonary bypass time increases the probability of complications such as hemolysis, coagulopathy, and inflammatory reactions [25]. Further, it means that the re-heparinization is finally exposed to the risk of PIH because it must be antagonized by the administration of protamine again. Less invasive treatments are required for this life-threatening condition. Additionally, there are also concerns regarding the dose of protamine. In the human medical setting, since refinement of protamine dosage is proposed [26], we also need to reconsider the protamine dosage in the veterinary medical setting. Lower doses of protamine may reduce the incidence of PIH, however further clinical research is needed.

## 5. Conclusions

Infusion of protamine effectively antagonized the anticoagulation but was associated with severe hypotension in two dogs. Administration of epinephrine was an effective treatment for hypotension in one dog but not in a second dog. Cardiopulmonary bypass was reinstated in the second dog, which was subsequently successfully weaned to spontaneous circulation. Epinephrine and extracorporeal hemodynamic support can be an effective strategy for catastrophic hypotension during protamine infusion.

## Figures and Tables

**Table 1:** Clinical characteristics, ACVIM stage, concurrent disease, and perioperative information of all dogs.

Variables	Non-Hypotensive (*n* = 12)	Hypotensive (*n* = 2)	
		Dog 1	Dog 2
Mean of Age, y	10.2 ± 1.5 (8.5–14)	11	9
Sex	cM (4), sF (4), M (3), F (1)	cM	cM
Breed	Chihuahua (5), Mongrel (2), Chin (1), CKCS (1), Maltese (1), Pomeranian (1), Shih Tzu (1)	CKCS	Chihuahua
Body weight, kg	4.3 ± 3.7 (2.4–8.0)	7	3.6
ACVIM Stage	B2 (5), C (4), D (3)	C	C
Blood pressure			
Before operation			
Systolic, mmHg	140 ± 23	156	146
Mean, mmHg	110 ± 30	110	109
Diastolic, mmHg	95 ± 30	87	91
One day after the operation			
Systolic BP, mmHg	135 ± 11	122	143
Mean BP, mmHg	91 ± 12	106	122
Diastolic BP, mmHg	70 ± 25	99	112
Heart rate, bpm	150 ± 44	133	156
Concurrent Disease	None (6), Renal Failure (1), Epilepsy (1), Hydrocephalus (1), Hypothyroidism (1), Idiopathic Thrombocytopenia (1)	none	None
Hospitalization, days	6 (6–12)	6	6
Time between heparin and protamine, min	125 ± 53	145	120
Mortality	1/12 (8.3%)	0/2 (0%)	

CKCS, Cavalier King Charles spaniel; cM, castrated male; F, intact female; M, intact male; sF, spayed female. Continuous data are expressed as the mean ± standard deviation (SD).

**Table 2:** Conventional echocardiographic variables before mitral valve surgery.

Variables	Unit	Non-Hypotensive (*n* = 12)	Hypotensive (*n* = 2)	
			Dog 1	Dog 2
BW	kg	4.38 ± 1.5	8	3.55
LVIDd	mm	33.4 ± 2.3	43	26.3
LVIDDN		2.2 ± 0.12	2.32	2.02
LA/Ao		2.55 ± 0.45	2.8	2.14
FS (%)	%	52.6 ± 6.5	56	62.7
E velocity	cm/s	144.9 ± 23.2	175	131
E/A		2.2 ± 0.9	5.14	1.32
S’ sep	cm/s	9.3 ± 1.5	9.1	10.9
E’ sep	cm/s	10.4 ± 2.6	7	10
A’ sep	cm/s	6.4 ± 1.5	7	7.3
S’ lat	cm/s	10.8 ± 3.0	6.1	10.3
E’ lat	cm/s	8.3 ± 2.0	7.2	9.1
A’ lat	cm/s	8.7 ± 3.6	5.3	8.9

Abbreviations: BW, body weight; LVIDd, left ventricular internal dimension in diastole; LVIDDN, normalized left ventricular internal dimension in diastole; LA/Ao, the ratio of the left atrial dimension to the aortic annulus dimension; FS, fractional shortening; E velocity, early diastolic mitral inflow velocity; E/A, the ratio of peak velocity of early diastolic transmitral flow to peak velocity of late diastolic transmitral flow; E’, early diastolic wave signal as measured by Tissue Doppler imaging (TDI); A’, late diastolic wave signal as measured by TDI; S’, systolic wave signal as measured by TDI; lat, mitral annulus at the left ventricular lateral wall; sep, mitral annulus at the septal wall.

**Table 3:** Catecholamine dose during protamine infusion.

Variables	Non-Hypotensive (*n* = 12)	Hypotensive (*n* = 2)		
		Dog 1	Dog 2	
Norepinephrine, μg^–1^ kg^–1^ min	0.05 (0–0.1)	0.1	0.2	
Dobutamine, μg^–1^ kg^–1^ min	4 (0–5)	5	5	

**Table 4:** Hemodynamic variables in non-hypotensive dogs (*n* = 12) after protamine infusion.

Variables		Time (min)										
		−10	−5	0	5	10	15	20	25	30	35	40
HR	bpm	102.6 (26.7)	102.3 (31.6)	104 (24)	106.3 (31.7)	98.4 (22.5)	97.6 (21.2)	93 (25.3)	97.8 (28.9)	94.7 (26.7)	87.9 (20.4)	90.5 (21.4)
SYS	mmHg	90.5 (14.9)	95.5 (17.8)	86.6 (13.8)	101.3 (24.3)	100.2 (17.4)	101.1 (20.5)	100.3 (16.1)	99.8 (19.8)	106.8 (19.4)	104.9 (17.3)	109.2 (26.3)
DIA	mmHg	59.9 (14.5)	59.4 (11.5)	55.7 (12.3)	61.5 (15.4)	62.7 (10.6)	59.7 (11)	60.4 (8.6)	60.3 (8.2)	63.6 (7.2)	62.9 (5.6)	65.9 (15.4)
MAP	mmHg	69 (12.9)	70.5 (11.5)	66.3 (13)	74.8 (17.7)	74.6 (10.9)	73.1 (12.7)	74.1 (9.1)	75.1 (10.4)	78.8 (9.3)	78.7 (8.5)	81.6 (16.3)
CVP	mmHg	8.3 (3.6)	7.2 (4.1)	7.1 (3.9)	7.8 (4.9)	7.5 (3.7)	7.2 (3.5)	6.4 (3.8)	6.5 (4.2)	5.9 (4.3)	6.9 (4.1)	7.4 (3.9)

Data are presented as mean (SD). Variables were reported every 5 min starting from 10 min before the start of protamine infusion to 40 min after the start of administration. Dunnett’s test was used to compare values for variables during protamine infusion with values before protamine infusion in program R (version 3.3.2; R Development Core Team 2016), with a significance set at *p* < 0.05. No significant change was observed in all variables. HR, heart rate; SYS, systolic arterial blood pressure; DIA, diastolic arterial blood pressure; MAP, mean arterial blood pressure; CVP, central venous pressure.

## Data Availability

The data are available through contact with the corresponding authors.

## Supplementary Materials

The following supporting information can be downloaded at: https://www.mdpi.com/article/10.3390/vetsci9040178/s1, Figure S1: Schematic illustration of the used procedures during the study.
