# Supplementary figures and images for: Hemodynamic Effects of Protamine Infusion in Dogs with Myxomatous Mitral Valve Disease Undergoing Mitral Valvuloplasty
Source: Vet Sci. 2022 Apr 8;9(4):178. doi: 10.3390/vetsci9040178 (PMC9031179; doi:10.3390/vetsci9040178)

(A)

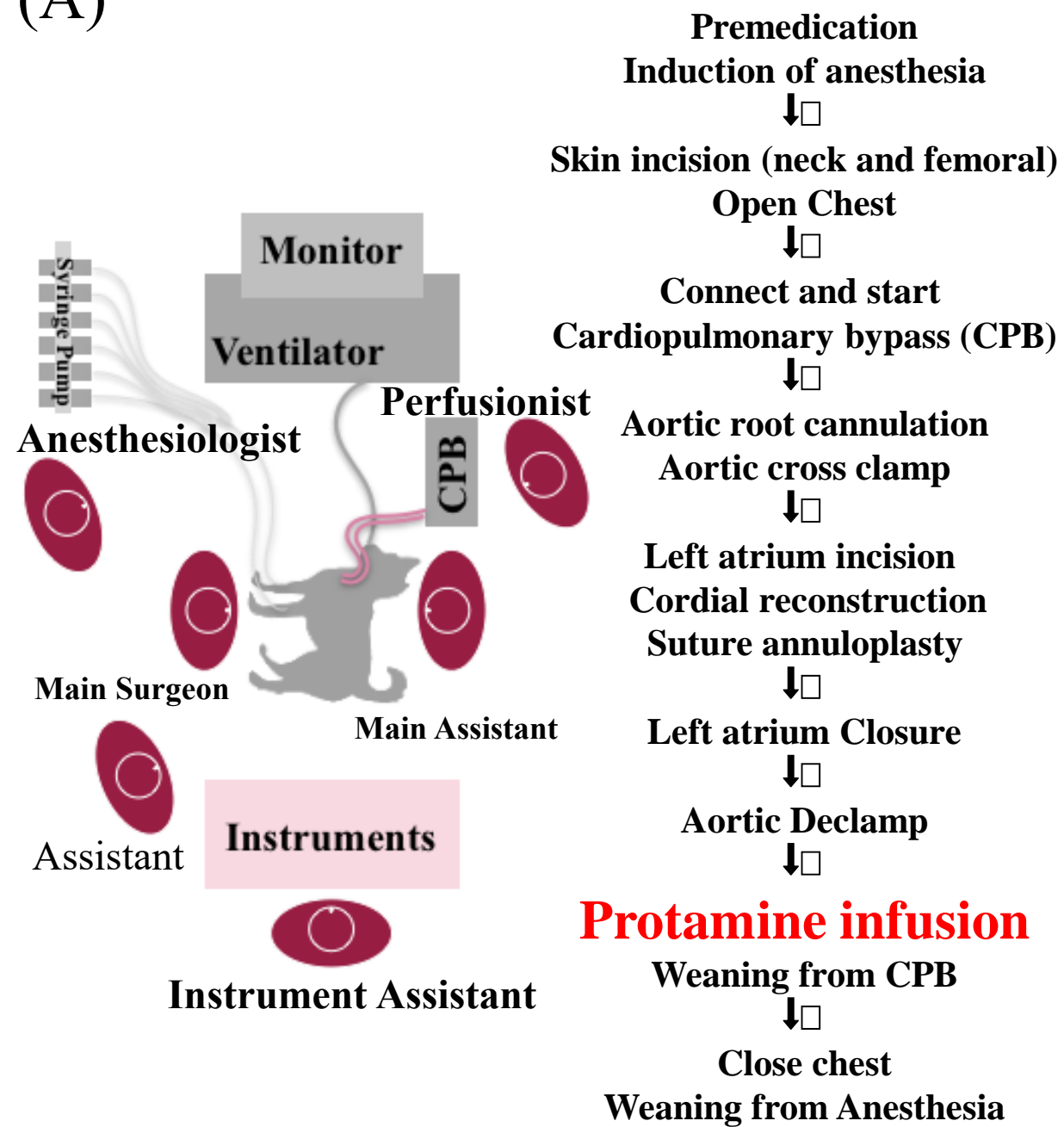

(B)

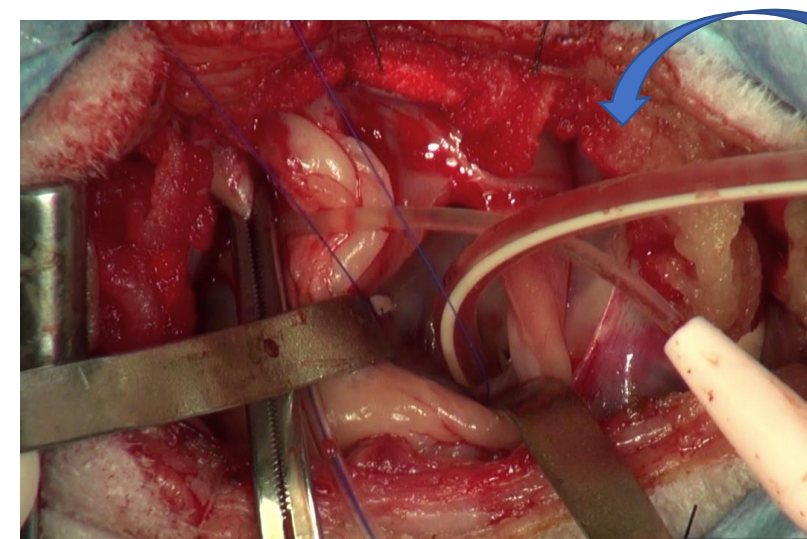

Suture annuloplasty

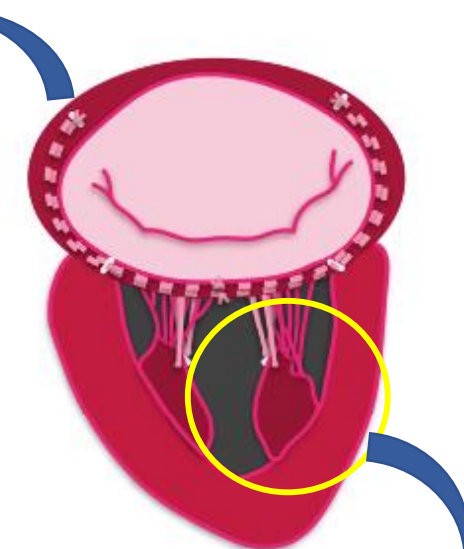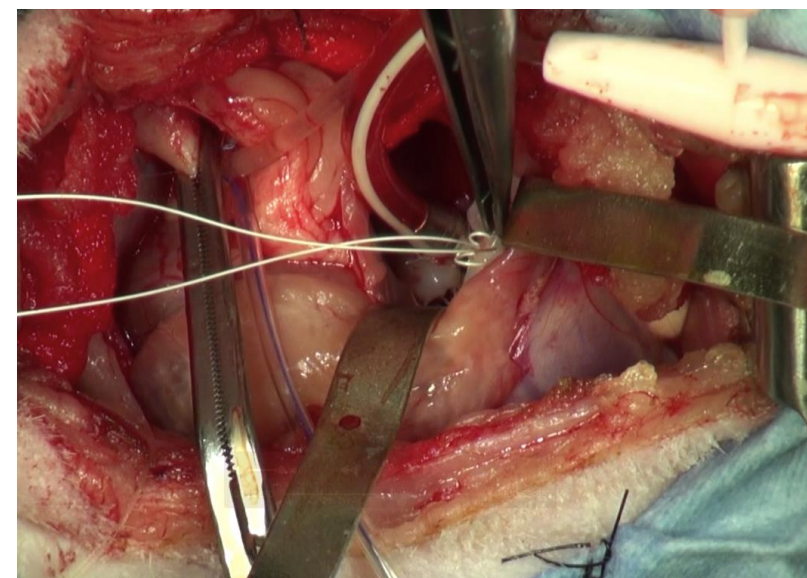

Cordial reconstruction

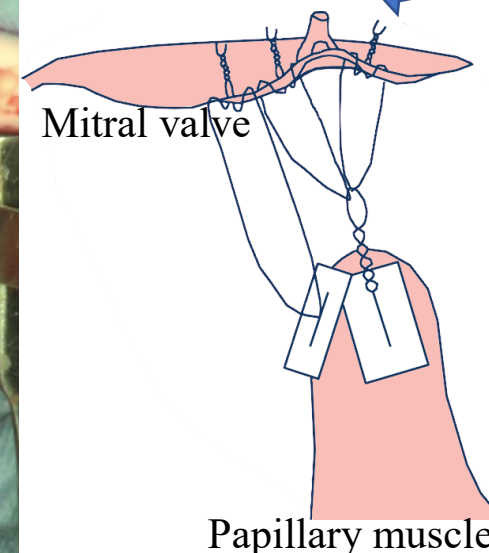

Supplement: Supplementary file 1 [file vetsci-09-00178-s001.zip › vetsci-1628648-supplementary Figure S1.pdf]
